# Mitigating Interfacial Mismatch between Lithium Metal and Garnet-Type Solid Electrolyte by Depositing Metal Nitride Lithiophilic Interlayer

**DOI:** 10.1021/acsaem.1c03157

**Published:** 2022-01-07

**Authors:** Abiral Baniya, Ashim Gurung, Jyotshna Pokharel, Ke Chen, Rajesh Pathak, Buddhi Sagar Lamsal, Nabin Ghimire, Raja Sekhar Bobba, Sheikh Ifatur Rahman, Sally Mabrouk, Alevtina L. Smirnova, Kang Xu, Quinn Qiao

**Affiliations:** † Mechanical and Aerospace Engineering, Syracuse University, Syracuse, New York 13244, United States; ‡ Department of Electrical Engineering and Computer Science, South Dakota State University, Brookings, South Dakota 57007, United States; § Applied Materials Division, Argonne National Laboratory, Lemont, Illinois 60439, United States; ∥ Department of Chemistry and Applied Biological Sciences, South Dakota School of Mines and Technology, Rapid City, South Dakota 57701, United States; ⊥ Battery Science Branch, Sensor and Electron Devices Directorate, U.S. Army Research Laboratory, Adelphi, Maryland 20783, United States

**Keywords:** solid-state electrolytes, lithium/garnet interface, interfacial resistance, solid-state batteries, silicon nitride

## Abstract

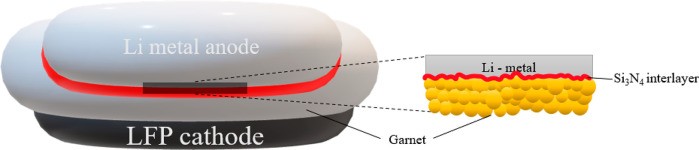

Solid-state lithium batteries are generally considered as the next-generation battery technology that benefits from inherent nonflammable solid electrolytes and safe harnessing of high-capacity lithium metal. Among various solid-electrolyte candidates, cubic garnet-type Li_7_La_3_Zr_2_O_12_ ceramics hold superiority due to their high ionic conductivity (10^–3^ to 10^–4^ S cm^−1^) and good chemical stability against lithium metal. However, practical deployment of solid-state batteries based on such garnet-type materials has been constrained by poor interfacing between lithium and garnet that displays high impedance and uneven current distribution. Herein, we propose a facile and effective strategy to significantly reduce this interfacial mismatch by modifying the surface of such garnet-type solid electrolyte with a thin layer of silicon nitride (Si_3_N_4_). This interfacial layer ensures an intimate contact with lithium due to its lithiophilic nature and formation of an intermediate lithium–metal alloy. The interfacial resistance experiences an exponential drop from 1197 to 84.5 Ω cm^2^. Lithium symmetrical cells with Si_3_N_4_-modified garnet exhibited low overpotential and long-term stable plating/stripping cycles at room temperature compared to bare garnet. Furthermore, a hybrid solid-state battery with Si_3_N_4_-modified garnet sandwiched between lithium metal anode and LiFePO_4_ cathode was demonstrated to operate with high cycling efficiency, excellent rate capability, and good electrochemical stability. This work represents a significant advancement toward use of garnet solid electrolytes in lithium metal batteries for the next-generation energy storage devices.

## 1 Introduction

Currently, lithium-ion batteries (LIBs) are used worldwide as the workhorse for powering applications.^1,2^ The ceiling of energy density allowed by commercial intercalation chemistries approaches 300 Wh/kg, while any attempt to push the energy density higher must face the risks imposed by highly flammable organic electrolyte solvents. Replacing graphite with lithium metal (Li^0^) as anode presents an ultimate solution, since lithium combines high specific capacity (3860 mAh g^–1^) with the lowest reduction potential (−3.04 V vs Li/Li^+^) among all elements in the Periodic Table.^3^ However, such low potential also makes lithium extremely reactive when in contact with almost any liquid electrolyte component. Liquid electrolytes also impose limitations on performance of high-voltage cathodes, due to their lower anodic stability.^4^ Therefore, development of high-energy and safe battery technologies relies on the replacement of liquid electrolytes with a fast ion conductor that does not combust. Solid-state batteries (SSBs) employing solid-state electrolytes (SSEs) hold such promises for the next-generation energy storage devices as long as they could be stable in the presence of both lithium and high-voltage cathode while conducting ions at fast rate.^5,6^

Several solid-electrolyte systems have been thoroughly explored, which range from sulfides to oxides and oxynitrides such as perovskite,^7^ antiperovskite,^8^ LISICON,^9^ thio-LISICON,^10^ NASICON,^11^ garnet,^12^ sulfide glass ceramic,^13−15^ etc. Certain sulfide SSEs (e.g., LGPS) are known for their ionic conductivity above 1 mS cm^–1^ at room temperature, but their sulfide nature renders them to be thermodynamically unstable against Li^0^ or high-voltage cathodes,^16−19^ while electrolytes, such as LIPON^20,21^ and LATP,^22,23^ also tend to react with Li^0^ anode (e.g., Ti^4+^/Ti^3+^ redox reaction). Only garnet SSEs, represented by Li_7_La_3_Zr_2_O_12_ (LLZO), provides high ionic conductivity close to 1 mS cm^–1^ at room temperature, a wide electrochemical window, and good electrochemical stability against Li^0^ anode.^24,25^

However, a major hurdle for garnets still exists: its poor contact with Li^0^, which arises from the microscopic gaps that are prevalent at solid–solid interfaces, and always leads to high interfacial impedance and poor cycling performance. Diversified strategies^26^ such as altering the chemical composition of the electrolyte,^27^ applying external heat and pressure,^28^ electrolyte surface modification,^29^ and interface modification^30^ have been adopted, among which the introduction of a buffer layer between garnet SSEs and Li^0^ has been proven efficient and promising. Buffer layers in the form of metals (such as Au,^31^ Al,^32^ Si,^33^ Ge,^34^ Mg^35^), metal oxides (such as Al_2_O_3_,^36^ ZnO^37^), and carbon material (such as graphite^38^) have significantly reduced impedance and improved cell performances. Computational analysis has revealed that material stability against Li^0^ depends on their cation and anion chemistry.^39^ Upon contact with Li^0^ these oxides, sulfides, and fluorides usually become unstable, which leads to the formation of an interlayer that consumes active materials and serves as a physical barrier to ion transport. Hence, metal nitrides are preferred as they are more stable against Li^0^ than oxides, sulfides, and fluorides.^39^

Here, we report a novel nitride interface modifier by coating the garnet-type Li_6.25_Al_0.25_La_3_Zr_2_O_12_ (Al-LLZO) solid electrolyte with a thin layer of Si_3_N_4_ deposited by radio frequency (RF) sputtering technique. This interfacial buffer layer enabled establishment of a homogeneous and intimate physical contact between the SSE and Li^0^. Thus, the developed nitride interface, denoted as Si_3_N_4_@Al-LLZO, showed a stable interface during cycling of symmetrical cells for a prolonged period of more than 800 h. With optimization of the Si_3_N_4_@Al-LLZO interfacial layer, Li/Si_3_N_4_@Al-LLZO/LFP full cells showed excellent overall cycling and rate performance.

## 2 Experimental Section

### 2.1 Garnet Al-LLZO Solid-Electrolyte Pellets Preparation

A 0.4 g amount of cubic phase aluminum doped lithium lanthanum zirconate garnet nanopowder, Li_6.25_Al_0.25_La_3_Zr_2_O_12_ (Ampcera Inc., 99.9%), was pressed into pellet by using 1/2 in. dry pellet pressing die (MTI Corp.) and applying 80 MPa pressure using a hydraulic laboratory press (Carver Inc.). Thus, obtained pellets were carefully placed on a magnesium oxide (MgO) crucible, covered with same mother powder and sintered in a furnace (Mellen, Microtherm) at 1280 °C for 1 h. After the pellets were left to cool to room temperature, they were dry polished from 1000, 1500, and 2000 to 3000 grit sized sandpapers using a rotary tool set (Fire Mountain Gems and Beads, USA). The polished pellets were stored in an argon glovebox for future use.

### 2.2 Si_3_N_4_ Interfacial Layer Deposition

Thin films of Si_3_N_4_ were deposited on polished Al-LLZO pellets using RF sputtering. A 2 in. diameter × 0.125 in. thick, 99.9% metals basis, silicon(IV) nitride (Si_3_N_4_) sample with MgO binder (Alfa Aesar) was used as target. The sputtering process was carried out at a deposition rate of 0.1 Å s^–1^ with 50 sccm constant flow of argon (Ar) gas. Various thicknesses (20, 30, and 40 nm) of Si_3_N_4_ thin films were investigated, and the thickness was optimized to 30 nm.

### 2.3 Solid-State Lithium Symmetrical Cells and Hybrid Solid-State Full Cells Assembly

First, for analyzing the ionic conductivity and cycling stability of as-prepared solid electrolytes, Li/Si_3_N_4_/Al-LLZO/Si_3_N_4_/Li symmetric cells were prepared by attaching the melted Li at 200 °C on both sides of the electrolyte pellets. After natural cooling, the Li/Si_3_N_4_/Al-LLZO/Si_3_N_4_/Li sample was assembled into coin cells in an argon-filled glovebox. Control symmetric cells without interface modification were also assembled for comparison with the modified one. Second, for preparation of Li/Si_3_N_4_@Al-LLZO/LFP hybrid solid-state full cells, the as-prepared Li/Si_3_N_4_@Al-LLZO sample was assembled with LiFePO_4_ (LFP) as cathode in a coin cell. For this, the cathode slurry was prepared by mixing LFP powders with Super-P carbon black and poly(vinylidene fluoride) (PVDF) at the weight ratio of 80:10:10, respectively, in *N*-methyl-2-pyrrolidone (NMP) solvent, using mortar and pestle. The as-prepared slurry was coated onto an aluminum foil and then dried in a vacuum oven at 120 °C overnight for thorough evaporation of the solvent. The dried cathode strips were then punched into circular disks with the active materials mass loading of ∼2 mg cm^–2^. Lastly, for assembly of hybrid solid-state full cell a tiny amount of 10 μL of liquid electrolyte (1.0 mol L^–1^ LiPF_6_ dissolved in ethylene carbonate (EC) and diethyl carbonate (DEC) in volume ratio of 1:1) was introduced between LFP cathode and solid-electrolyte pellet to enhance the cathode/electrolyte interface contact. The other side of the Al-LLZO pellet with no trace of liquid electrolyte was modified by Si_3_N_4_ deposition, and melted lithium was soldered on the top of it. The as-assembled full cell was sealed in a 2032 coin cell with nickel foam on the top for absorbing the excess pressure during crimping and avoiding damage to the solid-electrolyte pellet. The assembly of symmetric cells and full cells was done inside an argon-filled glovebox with moisture and O_2_ levels < 1 ppm.

### 2.4 Material Characterizations

The crystal structure of the samples was examined by X-ray diffraction (XRD) using a Rigaku SmartLab diffractrometer with Cu Kα radiation (λ = 1.54178 Å). Surface topography of bare garnet and Si_3_N_4_-modified garnet pellets were measured by an Agilent SPM 5500 atomic force microscope that is equipped with a MACIII controller and a RTESPA-525 tip with resonance frequency of 75 kHz. To observe the morphology of the samples, scanning electron microscopy (SEM) characterization was carried out using a Hitachi S-4300N scanning electron microscope, which was also equipped with energy-dispersive spectroscopy (EDS). Electrochemical impedance spectroscopy (EIS) measurement was done using the Ametek VERSASTAT3-200 potentiostat electrochemical workstation. The measurement was performed over a working frequency range of 1 MHz to 100 mHz with an amplitude of 10 mV. To measure the ionic conductivity of an Al-LLZO garnet-type pellet, 20 nm of gold (Au) layers were sputtered on both sides of the pellet as blocking electrode. Galvanostatic charge/discharge measurements of assembled coin cells were performed using aa LAND CT2001A system. The full cells were cycled at various current densities (e.g., 1C = 170 mA g^–1^) in a voltage range of 4.0 to 2.5 V. The coin cells were tested at room temperature.

## 3 Results and Discussion

### 3.1 Structure, Composition, and Kinetics of Prepared SSE

**Figure 1:**
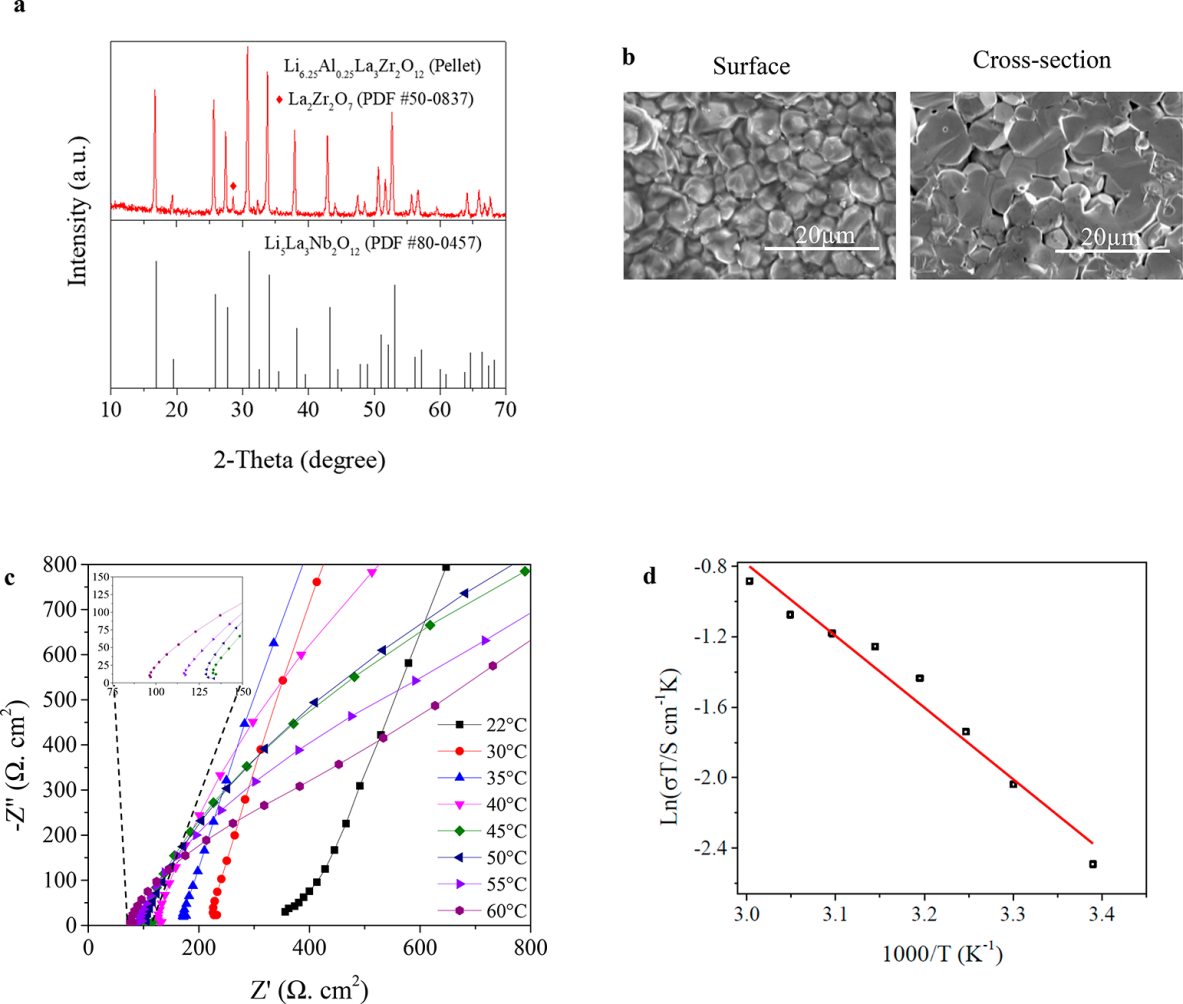
Characterization of as-prepared Al-LLZO garnet electrolyte pellet. (a) XRD comparison of Al-LLZO garnet pellet that matches with cubic structure Li_5_La_3_Nb_2_O_12_. (b) Surface and cross-section SEM images of Al-LLZO pellets. (c) EIS spectra of Al-LLZO electrolyte at elevated temperatures ranging from 22 to 60 °C. Inset showing spectra from 45 to 60 °C. (d) Arrhenius plot of Al-LLZO ionic conductivity.

The cubic phase Li_6.25_Al_0.25_La_3_Zr_2_O_12_ garnet nanopowder was pressed, sintered, and polished into solid-electrolyte pellets (Supporting Information Figure S1). As shown in Figure S2c, first, XRD was performed on a polished pellet, pressed using as-received Al-LLZO powder and sintered at 1000 °C. The impurity peaks, marked with an asterisk (*), were identified as La_2_Zr_2_O_7_ (PDF No. 50-0837). Further, when the garnet nanopowder was pressed, sintered (>1200 °C), and polished, most of the impurity peaks of La_2_Zr_2_O_7_ disappeared and the one at about 29° was much suppressed, marked by a red diamond, which indicated formation of a rather pure cubic phase Al-LLZO garnet pellet. Also, XRD patterns (Figure 1a) of as-prepared solid-electrolyte pellets show the resemblance of diffraction peaks when indexed to the standard pattern of cubic garnet phase Li_5_La_3_Nb_2_O_12_ (PDF No. 80-0457). Further, the surface and cross-section SEM images (Figure 1b) show well-densified pellets with the majority of grains tightly connected when sintered at 1280 °C for an hour. These sintered pellets have relative densities of ∼92% (Figure S2) when measured using Archimedes’ principle and ethanol as immersion medium.^40^

The ionic conductivities of Al-LLZO pellets were evaluated using EIS with Au layers as blocking electrodes. The total Li-ion conductivity of Al-LLZO pellets using the low-frequency intercept value was calculated to be 2.81 × 10^–4^ S cm^–1^. The Li-ion conductivity of Al-LLZO was also measured at temperatures ranging from 22 to 65 °C (Figure 1c), where the low-frequency intercept value decreases (Figure S3c) by following typical Arrhenius behavior (Figure 1d). Activation energy (*E*_a_) for Li-ion conduction was calculated using eq 1:
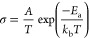
1where *A* is a pre-exponential factor, *E*_a_ is the activation energy, *k*_b_ is the Boltzmann constant, and *T* is the absolute temperature. Thus, observed activation energy and Li-ion conductivity of 0.34 eV and 2.81 × 10^–4^ S cm^–1^ at 22 °C, respectively are in line with other reports for garnet SSE.^41−43^

### 3.2 Metal Nitride Interface Layer Properties

**Figure 2:**
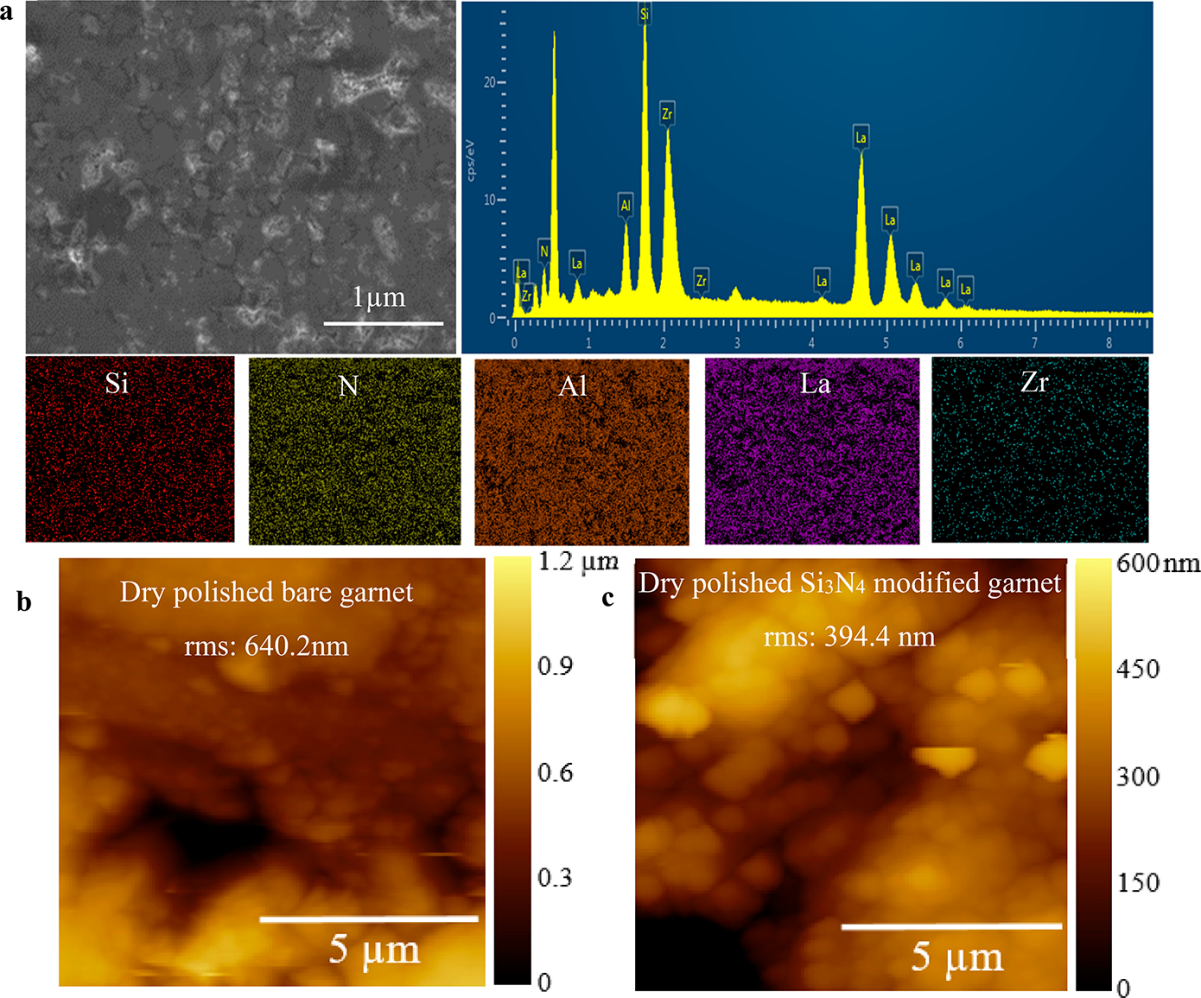
EDS spectrum and AFM mapping of bare and Si3N_4_-modified Al-LLZO garnet pellet SSE surface. (a) EDS spectrum shows presence of Si and N along with elements from SSE. AFM topography mapping of dry polished (b) bare garnet and (c) Si_3_N_4_-modified garnet.

The improved interfacial contact between Li^0^ and Al-LLZO garnet electrolyte is crucial for enhanced ion transport and even current distribution at the interface. However, the contact between Li^0^ and bare garnet consists of voids and gaps leading to uneven current distribution at the interface that accelerates dendrite or dead Li^0^ growth that could short circuit through the solid electrolyte. To address this issue, a thin film of Si_3_N_4_ was sputter deposited on top of an Al-LLZO garnet pellet. Figure 2a shows the energy-dispersive X-ray spectroscopy (EDS) spectrum and mapping of a Si_3_N_4_-deposited Al-LLZO garnet pellet, which reveals the presence of La, Zr, and Al in the garnet along with N and Si attributed to the deposited Si_3_N_4_. Further, atomic force microscopy (AFM) performed on bare (Figure 2b) and Si_3_N_4_-modified (Figure 2c) garnet samples compares their surface roughness using the average surface root-mean-square (RMS) values, which reveals the presence of Si_3_N_4_ significantly reduces the RMS value from 640.2 nm of bare garnet to 394.4 nm. The higher RMS value represents the uneven and rough surface of dry polished bare garnet that leads to poor contacts^44^ and induces uneven current distribution^45,46^ that eventually leads to preferential deposition of Li^0^^47^ on certain spots and formation of dendrites.^48^ The lower RMS value of Si_3_N_4_-modified dry polished garnet should result in much more uniform and stable Li plating/stripping that is conducive for longer cycling life.^49^

**Figure 3:**
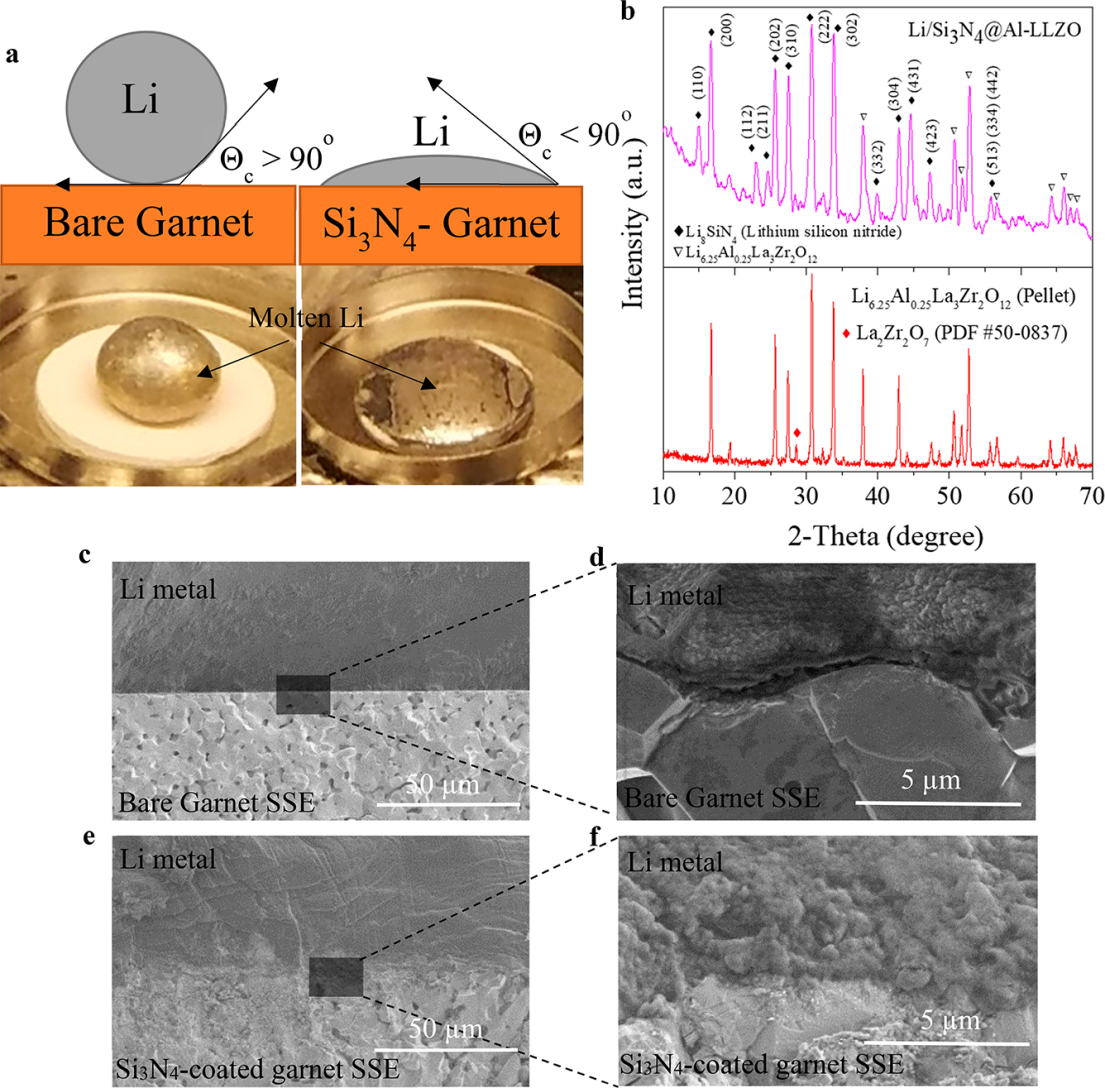
Wetting behavior and interfacial contact characterization of Li| garnet SSE and Li|Si_3_N_4_-coated garnet SSE. (a) Digital images of bare Al-LLZO garnet pellet with molten Li on top with contact angle (θc > 90°), and Si_3_N_4_-deposited Al-LLZO pellet with molten Li on top with contact angle (θ*c* < 90°). (b) XRD comparison of thus prepared bare garnet and Si_3_N_4_-coated garnet. Cross-section SEM images of Li/Al-LLZO interface (c, d) without and (e, f) with a Si_3_N_4_ interlayer.

After Si_3_N_4_ thin film deposition, as shown in SEM images of Figure 3e,f, the Li^0^ anode has been tightly soldered with an Al-LLZO pellet as no gaps and voids are visible in comparison to bare garnet (Figure 3c,d). This depicts that the Si_3_N_4_ thin film at the interface enabled the promotion of interfacial contact of Al-LLZO grains with lithium metal. To observe the lithiophilicity of the Si_3_N_4_ interfacial layer, a molten Li^0^ droplet was applied to the bare and Si_3_N_4_-coated garnet pellets, respectively. As observed from Figure 3a, the molten Li^0^ on the top of the bare garnet pellet instantly beads up to form a ball, revealing its lithiophobicity. In contrast, with the Si_3_N_4_-coated garnet, the molten lithium readily wets the surface and spreads out to fully cover it.

To further demonstrate this conversion of lithiophobicity to lithiophilicity, Li^0^ foil was gradually heated on the top of the Si_3_N_4_-coated garnet surface. As shown in Figure S4, when Li^0^ starts to melt at ∼190 °C, the Si_3_N_4_-coated area in proximity to lithium metal turns black in color,, which suggests occurrence of lithiation reaction of as-deposited Si_3_N_4_. This reaction not only occurred at the areas directly under lithium metal but also around the entire Si_3_N_4_-coated garnet.

XRD was performed after Si_3_N_4_ deposition on an SSE pellet and infusing molten lithium on top of it, Figure 3b shows the appearance of some new peaks indicated as black-filled diamonds along with the common diffraction peaks related to Al-LLZO. These pronounced new peaks indicate the formation of tetragonal phase Li_8_SiN_4_, lithium silicon nitride (JCPDS Card No. 40-1449)^50,51^ when lithium reacts with the silicon nitride layer at the interface and can also be verified from previous literature.^52−59^ The formation of ternary alloy phase Li_8_SiN_4_ is further explained in Figure 3b by identifying peaks using Miller indices. These peaks match the XRD data that are reported by Yamashita et al. in ref (60), by Yamane et al. in ref (52), and from JCPDS Card No. 40-1449. These alloys at the interface provide open tunnels for Li^+^ conduction as all phases of these alloys are shown to conduct Li^+^ where a phase such as Li_8_SiN_4_ can show conductivity reaching as high as 5 × 10^–2^ S m^–1^ at 400 K with lowest activation energy (46 kJ/mol).^52^ Studies by Yamane et al.^52^ and Ulvestad et al.^53^ have shown the thermal formation of different ternary lithium silicon nitrides from Si_3_N_4_ when in contact with Li^0^. Heating was provided during infusion of molten Li^0^ in Si_3_N_4_ layer which further assists in formation of ternary phase alloy. These alloys are very ionically conductive, which is self-evident by the decrease in interfacial and charge transfer resistance by introduction of the Si_3_N_4_ interlayer. On the basis of this hypothesis, chemical eq 2 can best describe the initial reduction reaction:^53^
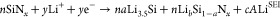
2Thus, the conversion reaction of Si_3_N_4_ film deposited at the interface with Li^0^^38^ results in formation of ternary phase alloy, e.g., Li_8_SiN_4_,^52^ which enhances the interfacial contact.

Furthermore, coating amorphous silicon (Si) atoms have been known to switch the surface of garnet LLZO from “superlithiophobic” to “superlithiophilic”.^33^ Similarly, lithium nitride (Li_3_N) in cases of both garnet solid^61^ and carbonate based liquid electrolyte^62^ have been shown to drastically decrease the interfacial impedance and passivate the surface of Li anode. On the basis of these previous findings, silicon nitride (Si_3_N_4_) is propitious to show both strong wetting interaction with molten Li^0^ due to the presence of nitride that undergoes alloying reaction.

### 3.3 Electrochemical Properties of Interface Stabilized SSE

**Figure 4:**
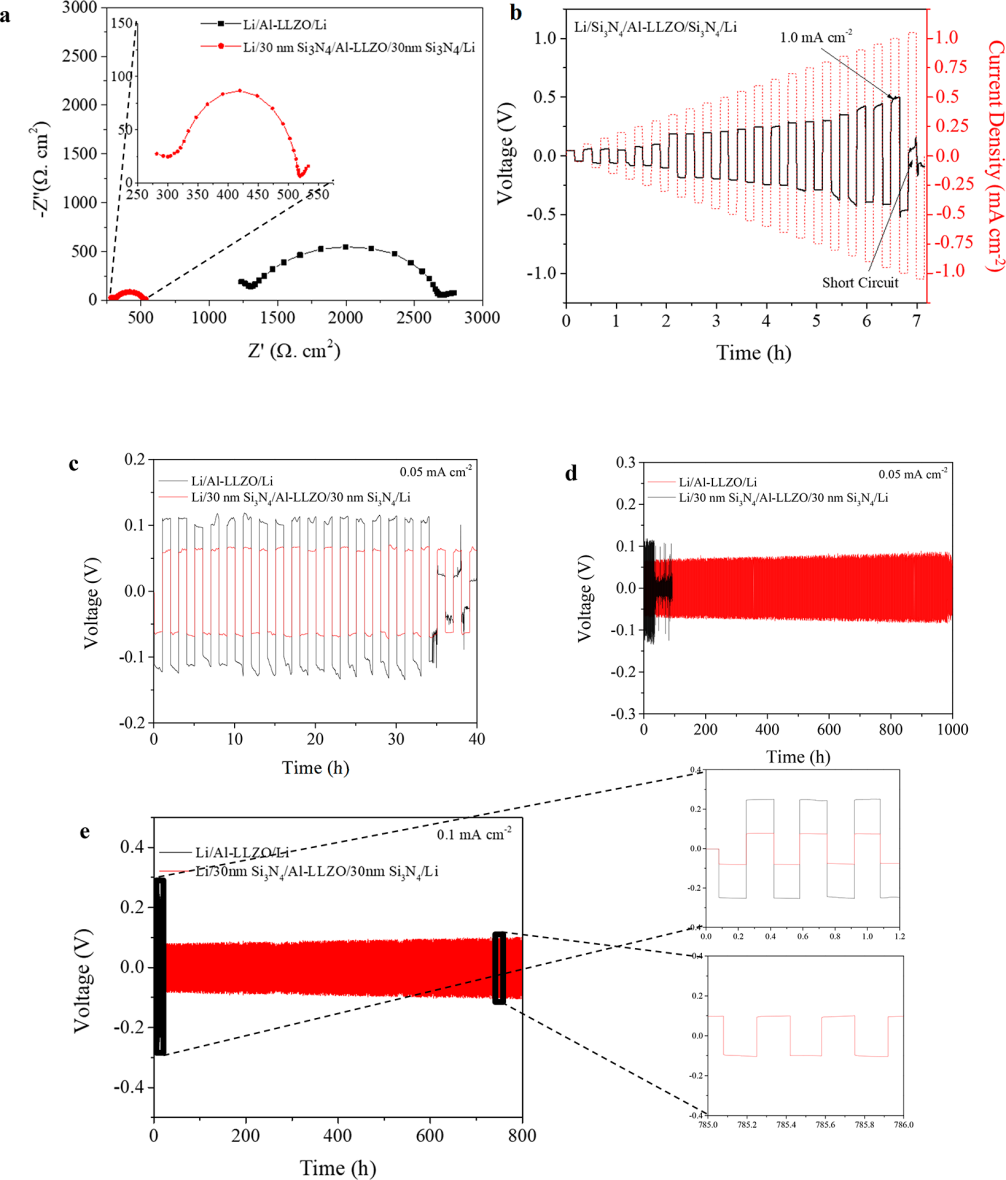
Electrochemical stability of interface-modified SSE. (a) Nyquist plots of Li symmetrical cells for Al-LLZO with and without Si_3_N_4_ modification. (b) Critical current density (CCD) plot for Li/Si_3_N_4_/Al-LLZO/Si_3_N_4_/Li symmetric cell. Galvanostatic cycling performance of Li/Al-LLZO/Li symmetrical cells with and without Si_3_N_4_ modification at 0.05 mA cm^–2^ and 0.05 mAh cm^–2^. (c) First few cycles and (d) long-term cycling. (e) Galvanostatic cycling performance of Li/Si_3_N_4_/Al-LLZO/Si_3_N_4_/Li symmetric cell at constant current density of 0.1 mA cm^–2^.

Symmetric cells Li/Si_3_N_4_/Al-LLZO/Si_3_N_4_/Li and Li/Al-LLZO/Li were assembled and characterized, whose Nyquist plots (Figure 4a) show that the introduction of Si_3_N_4_ reduces total impedance (combined impedance of Al-LLZO electrolyte pellet and Li/Al-LLZO interface) from 2750 Ω cm^2^ for the bare garnet to 525 Ω cm^2^ for the modified one (Figure 4a). At 22 °C, the total impedance of the Au/Al-LLZO/Au sample was observed to be 356 Ω cm^2^ (Figure 1c). Judging from the values from Figure 4a for combined impedances, the interfacial ASR has been reduced from 1197 to 84.5 Ω cm^2^. Similarly, as shown in Figure 4b, the CCD of the Li/Si_3_N_4_/Al-LLZO/Si_3_N_4_/Li symmetric cell was tested and confirmed to be 1 mA cm^–2^. This significant reduction of interfacial ASR can be attributed to (1) the Si_3_N_4_ interlayer promoting conformal contact of Li^0^ anode on SSE; (2) formation of thermally lithiated Si_3_N_4_ when Li^0^ is heated in contact with the interlayer; and (3) inhibition of impurity layers, such as, Li_2_CO_3_ due to coating of Si_3_N_4_ on SSE surface.

Galvanostatic Li plating/stripping cycling experiments using Li symmetrical cells were performed to assess the effectiveness of Li-ion transport across the interface and cycling stability. For this, various thicknesses of Si_3_N_4_ (for example 20, 30, and 40 nm) interlayer were deposited on top of the garnet surface and it was optimized to 30 nm (Figure S5). As shown in Figure 4 and Figure S6, plating/stripping cycles of symmetrical cells were performed in both low and high current densities of 0.05 and 0.2 mA cm^–2^, respectively. Figure 4c shows comparison of the first few plating/stripping cycles of Li symmetrical cells based on bare garnet and Si_3_N_4_-modified garnet cycled at current density of 0.05 mA cm^–2^ and capacity of 0.05 mAh cm^–2^. It can be observed that the symmetric cell with bare garnet is plagued with large overpotential > ±100 mV, while the cell with the Si_3_N_4_ interface layer facilitated the suppression of this overpotential to ±60 mV. This indicates that the introduction of Si_3_N_4_ reduced the energy barrier of the lithium transfer process at the interface, thus facilitating the occurrence of efficient plating/stripping cycles. Longer plating/stripping cycling of these symmetrical cells was carried out as shown in Figure 4d. The cell with bare garnet short circuiting after only 35 h can be attributed to typical phenomenon of Li infiltration into SSE (Figure S6c).^63^ In contrast, the cell with Si_3_N_4_-modified garnet shows stable cycling for 1000 h, suggesting a stable interface enabled by Si_3_N_4_ thin film. Similar stable cycling up to 800 h at current density of 0.1 mA cm^–2^ was demonstrated by the Si_3_N_4_-modified garnet with voltage stabilized at ∼80 mV (as further indicated by voltage profiles in the inset of Figure 4e), while the cell with bare garnet could last only 20 h with large voltage polarization of ∼250 mV.

This excellent cycling with low-voltage polarization confirms the establishment of a stable interface with low interfacial impedance by introduction of the Si_3_N_4_ interfacial layer. Also, longer and stable cycling with almost unchanged polarization and overpotential of ∼100 mV was exhibited at higher current density of 0.2 mA cm^–2^ (Figure S6a,b). The prompt short circuiting of bare garnet compared to garnet with a Si_3_N_4_-modified interface shows that superior stability of the interface is attained by Si_3_N_4_ deposition. Comparison of the performance of Si_3_N_4_ interlayer in this work with other reported interlayers is summarized in Table S1. It can be noticed that, for room temperature (22 °C) operation, the Si_3_N_4_ interlayer shows remarkably low interfacial resistance at reduced voltage overpotential. Also, the critical current density of 1 mA cm^–2^ achieved is very much comparable considering the electrolyte thickness and deposition procedure employed in this work. These observations imply that Si_3_N_4_ coating as interlayer can homogenize current distribution at the Li/garnet interface by addressing the interface mismatch between Li-anode and SSE.

### 3.4 Full Cell Demonstration of Interface Stabilized SSE

**Figure 5:**
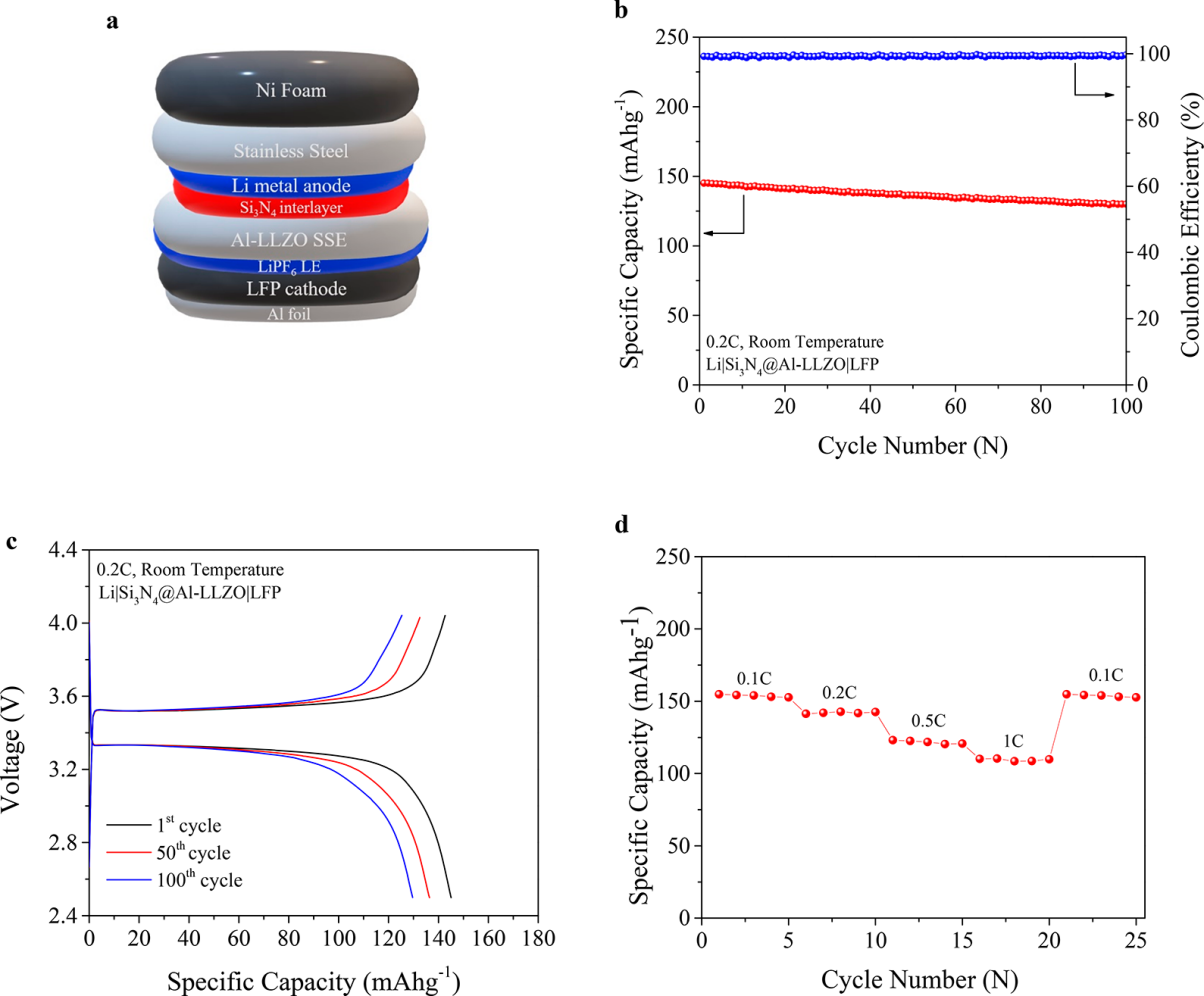
Full cell demonstration of electrochemical cells. (a) Schematic of device structure for Li/Si_3_N_4_@Al-LLZO/LFP cell. (b) Cycling performance of the cell at 0.2C-rate and room temperature. (c) Voltage profiles for selected cycles (first, 50th, and 100th) of Li/Si_3_N_4_@Al-LLZO/LFP cell at 0.2C and room temperature. (d) Rate performance of cell at different C-rates.

Further, to demonstrate the potential to enable high-energy density Li-metal batteries by the interface stability approach developed in this work, Li/Si_3_N_4_@Al-LLZO/LFP hybrid solid-state full cells as shown in Figure 5a were assembled and tested. The cathode/garnet interface (Si_3_N_4_@Al-LLZO/LFP) was wetted with a tiny amount of liquid organic electrolyte to reduce cathode/electrolyte interfacial resistance. The Li/Si_3_N_4_@Al-LLZO/LFP cells showed low charge transfer resistance (Figure S7) and stable cycling compared to Li/Al-LLZO/LFP cells (Figure S8). Figure 5b shows the galvanostatic charge/discharge cycling performance of the full cell with Si_3_N_4_@Al-LLZO garnet electrolyte at current density of 0.2C. The cell delivered initial charge and discharge capacities of 146.25 and 145.11 mAh g^–1^, respectively, that correspond to the Coulombic efficiency of 99.2%. The discharge capacity after 100 cycles was 130 mAh g^–1^ while maintaining the Coulombic efficiency close to 100%. As shown in Figure 5c, the full cell with Si_3_N_4_@Al-LLZO garnet electrolyte exhibits well-defined and flat voltage plateaus with small polarization of ∼0.15 V at first, 50th, and 100th cycles tested at 0.2C and room temperature. The Si_3_N_4_@Al-LLZO full cells were further cycled at various C-rates of 0.1, 0.2, 0.5, and 1C. As shown in Figure 5d, the cell demonstrated good rate capability with discharge capacities of 153.8, 142.1, 121.7, and 109.5 mAh g^–1^ obtained at 0.1, 0.2, 0.5, and 1C, respectively. The cell displayed discharge capacity retention of 153.8 mAh g^–1^ at 0.1C which accounted for ∼100% of the initial capacity after five cycles each of higher C-rates. These observations further validate the efficacy of introducing Si_3_N_4_ as Li/garnet interface modifier to obtain stable and high energy density solid-state Li-metal batteries.

## 4 Conclusions

The poor interfacing between Li^0^ and garnet-type Al-LLZO solid-state electrolyte by introducing a sputter-coated thin Si_3_N_4_ intermediate layer was addressed. The Si_3_N_4_ coating on the Al-LLZO solid-electrolyte pellet significantly reduces Li/Al-LLZO interfacial resistance from 1197 to 84.5 Ω cm^2^, promotes better wettability of Li^0^ with Al-LLZO electrolyte, and facilitates efficient charge transfer at the interface. Noticeably, symmetrical cells with much lower overpotential and long plating/stripping cycling for >800 h at current density of 0.1 mA cm^–2^ were demonstrated using the Si_3_N_4_-modified Al-LLZO solid electrolyte. Along with it, Si_3_N_4_@Al-LLZO solid electrolyte paired with Li^0^ as anode and LFP as cathode exhibited stable cycling performance with excellent Coulombic efficiency compared to that for bare garnet. Introduction of Si_3_N_4_ facilitated formation of lithiophilic interface which in turn contributed to establishment of an intimate and conformal physical/chemical contact between garnet-type solid electrolyte and lithium. The present work successfully resolves the primary challenge of high impedance Li/garnet-type solid-electrolyte interface for solid-state batteries. These findings can provide further insights into engineered interfaces focused on development of high energy density and safe solid-state Li-metal batteries.

## Supporting Information Available

Digital images of as-prepared Al-LLZO pellet; surface and cross-section SEM of pellets at different sintering temperatures; XRD and Raman showing impurity removal; EIS spectra at different temperatures; digital image of lithiation reaction; optimization of interlayer thickness; plating/stripping at 0.2 mA cm^–2^; Nyquist plots for full cells; full cell cycling; comparison table (PDF)

## References

[ref1] Armand M.; Tarascon J.-M. Building better batteries. nature 2008, 451 (7179), 652 10.1038/451652a. 18256660

[ref2] Peng L.; Zhu Y.; Chen D.; Ruoff R. S.; Yu G. Two-Dimensional Materials for Beyond-Lithium-Ion Batteries. Adv. Energy Mater. 2016, 6 (11), 1600025 10.1002/aenm.201600025.

[ref3] Xu W.; Wang J.; Ding F.; Chen X.; Nasybulin E.; Zhang Y.; Zhang J.-G. Lithium metal anodes for rechargeable batteries. Energy Environ. Sci. 2014, 7 (2), 513–537. 10.1039/C3EE40795K.

[ref4] Dunn B.; Kamath H.; Tarascon J.-M. Electrical energy storage for the grid: a battery of choices. Science 2011, 334 (6058), 928–935. 10.1126/science.1212741. 22096188

[ref5] Hu Y.-S. Batteries: Getting solid. Nat. Energy 2016, 1 (4), 16042 10.1038/nenergy.2016.42.

[ref6] Janek J.; Zeier W. G. A solid future for battery development. Nat. Energy 2016, 1 (9), 16141 10.1038/nenergy.2016.141.

[ref7] Ma C.; Chen K.; Liang C.; Nan C.-W.; Ishikawa R.; More K.; Chi M. Atomic-scale origin of the large grain-boundary resistance in perovskite Li-ion-conducting solid electrolytes. Energy Environ. Sci. 2014, 7 (5), 1638–1642. 10.1039/c4ee00382a.

[ref8] Dondelinger M.; Swanson J.; Nasymov G.; Jahnke C.; Qiao Q.; Wu J.; Widener C.; Numan-Al-Mobin A. M.; Smirnova A. Electrochemical stability of lithium halide electrolyte with antiperovskite crystal structure. Electrochim. Acta 2019, 306, 498–505. 10.1016/j.electacta.2019.03.074.

[ref9] Hong H.-P. Crystal structure and ionic conductivity of Li14Zn (GeO4) 4 and other new Li+ superionic conductors. Mater. Res. Bull. 1978, 13 (2), 117–124. 10.1016/0025-5408(78)90075-2.

[ref10] Kanno R.; Hata T.; Kawamoto Y.; Irie M. Synthesis of a new lithium ionic conductor, thio-LISICON–lithium germanium sulfide system. Solid State Ionics 2000, 130 (1–2), 97–104. 10.1016/S0167-2738(00)00277-0.

[ref11] Monchak M.; Hupfer T.; Senyshyn A.; Boysen H.; Chernyshov D.; Hansen T.; Schell K. G.; Bucharsky E. C.; Hoffmann M. J.; Ehrenberg H. Lithium diffusion pathway in Li1. 3Al0. 3Ti1. 7 (PO4) 3 (LATP) superionic conductor. Inorganic chemistry 2016, 55 (6), 2941–2945. 10.1021/acs.inorgchem.5b02821. 26930220

[ref12] Murugan R.; Thangadurai V.; Weppner W. Fast lithium ion conduction in garnet-type Li7La3Zr2O12. Angew. Chem., Int. Ed. 2007, 46 (41), 7778–7781. 10.1002/anie.200701144. 17803180

[ref13] Bron P.; Johansson S.; Zick K.; Schmedt auf der Gunne J.; Dehnen S.; Roling B. Li_10_SnP_2_S_12_: An affordable lithium superionic conductor. J. Am. Chem. Soc. 2013, 135 (42), 15694–15697. 10.1021/ja407393y. 24079534

[ref14] Deiseroth H. J.; Kong S. T.; Eckert H.; Vannahme J.; Reiner C.; Zaiß T.; Schlosser M. Li6PS5X: a class of crystalline Li-rich solids with an unusually high Li+ mobility. Angew. Chem., Int. Ed. 2008, 47 (4), 755–758. 10.1002/anie.200703900. 18161703

[ref15] Kamaya N.; Homma K.; Yamakawa Y.; Hirayama M.; Kanno R.; Yonemura M.; Kamiyama T.; Kato Y.; Hama S.; Kawamoto K.; et al. A lithium superionic conductor. Nat. Mater. 2011, 10 (9), 682–686. 10.1038/nmat3066. 21804556

[ref16] Li Y.; Zhou W.; Chen X.; Lü X.; Cui Z.; Xin S.; Xue L.; Jia Q.; Goodenough J. B. Mastering the interface for advanced all-solid-state lithium rechargeable batteries. Proc. Natl. Acad. Sci. U. S. A. 2016, 113 (47), 13313–13317. 10.1073/pnas.1615912113. 27821751 PMC5127322

[ref17] Wenzel S.; Leichtweiss T.; Krüger D.; Sann J.; Janek J. Interphase formation on lithium solid electrolytes—An in situ approach to study interfacial reactions by photoelectron spectroscopy. Solid State Ionics 2015, 278, 98–105. 10.1016/j.ssi.2015.06.001.

[ref18] Zhu Y.; He X.; Mo Y. Origin of outstanding stability in the lithium solid electrolyte materials: insights from thermodynamic analyses based on first-principles calculations. ACS Appl. Mater. Interfaces 2015, 7 (42), 23685–23693. 10.1021/acsami.5b07517. 26440586

[ref19] Zhu Y.; He X.; Mo Y. First principles study on electrochemical and chemical stability of solid electrolyte–electrode interfaces in all-solid-state Li-ion batteries. Journal of Materials Chemistry A 2016, 4 (9), 3253–3266. 10.1039/C5TA08574H.

[ref20] Bates J. B.; Dudney N. J.; Gruzalski G. R.; Zuhr R. A.; Choudhury A.; Luck C. F.; Robertson J. D. Electrical properties of amorphous lithium electrolyte thin films. Solid State Ionics 1992, 53-56, 647–654. 10.1016/0167-2738(92)90442-R.

[ref21] Bates J.B.; Dudney N.J.; Lubben D.C.; Gruzalski G.R.; Kwak B.S.; Yu X.; Zuhr R.A. Thin-film rechargeable lithium batteries. J. Power Sour. 1995, 54 (1), 58–62. 10.1016/0378-7753(94)02040-A.

[ref22] Aono H.; Sugimoto E.; Sadaoka Y.; Imanaka N.; Adachi G.y. Ionic conductivity of solid electrolytes based on lithium titanium phosphate. J. Electrochem. Soc. 1990, 137 (4), 1023–1027. 10.1149/1.2086597.

[ref23] Ding Z.; Li J.; Li J.; An C. Interfaces: Key issue to be solved for all solid-state lithium battery technologies. J. Electrochem. Soc. 2020, 167 (7), 070541 10.1149/1945-7111/ab7f84.

[ref24] Liu Q.; Geng Z.; Han C.; Fu Y.; Li S.; He Y.-b.; Kang F.; Li B. Challenges and perspectives of garnet solid electrolytes for all solid-state lithium batteries. J. Power Sources 2018, 389, 120–134. 10.1016/j.jpowsour.2018.04.019.

[ref25] Zhang X.; Liu T.; Zhang S.; Huang X.; Xu B.; Lin Y.; Xu B.; Li L.; Nan C.-W.; Shen Y. Synergistic coupling between Li6. 75La3Zr1. 75Ta0. 25O12 and poly (vinylidene fluoride) induces high ionic conductivity, mechanical strength, and thermal stability of solid composite electrolytes. J. Am. Chem. Soc. 2017, 139 (39), 13779–13785. 10.1021/jacs.7b06364. 28898065

[ref26] Gurung A.; Pokharel J.; Baniya A.; Pathak R.; Chen K.; Lamsal B. S.; Ghimire N.; Zhang W.-H.; Zhou Y.; Qiao Q. A review on strategies addressing interface incompatibilities in inorganic all-solid-state lithium batteries. Sustainable Energy & Fuels 2019, 3 (12), 3279–3309. 10.1039/C9SE00549H.

[ref27] Li Y.; Xu B.; Xu H.; Duan H.; Lü X.; Xin S.; Zhou W.; Xue L.; Fu G.; Manthiram A.; et al. Hybrid polymer/garnet electrolyte with a small interfacial resistance for lithium-ion batteries. Angew. Chem., Int. Ed. 2017, 56 (3), 753–756. 10.1002/anie.201608924. 27936306

[ref28] Cheng L.; Crumlin E. J.; Chen W.; Qiao R.; Hou H.; Lux S. F.; Zorba V.; Russo R.; Kostecki R.; Liu Z.; et al. The origin of high electrolyte–electrode interfacial resistances in lithium cells containing garnet type solid electrolytes. Phys. Chem. Chem. Phys. 2014, 16 (34), 18294–18300. 10.1039/C4CP02921F. 25057850

[ref29] Sharafi A.; Kazyak E.; Davis A. L.; Yu S.; Thompson T.; Siegel D. J.; Dasgupta N. P.; Sakamoto J. Surface chemistry mechanism of ultra-low interfacial resistance in the solid-state electrolyte Li7La3Zr2O12. Chem. Mater. 2017, 29 (18), 7961–7968. 10.1021/acs.chemmater.7b03002.

[ref30] Tsai C.-L.; Roddatis V.; Chandran C. V.; Ma Q.; Uhlenbruck S.; Bram M.; Heitjans P.; Guillon O. Li7La3Zr2O12 interface modification for Li dendrite prevention. ACS Appl. Mater. Interfaces 2016, 8 (16), 10617–10626. 10.1021/acsami.6b00831. 27029789

[ref31] Wakasugi J.; Munakata H.; Kanamura K. Effect of gold layer on interface resistance between lithium metal anode and Li6. 25Al0. 25La3Zr2O12 solid electrolyte. J. Electrochem. Soc. 2017, 164 (6), A1022 10.1149/2.0471706jes.

[ref32] Fu K. K.; Gong Y.; Liu B.; Zhu Y.; Xu S.; Yao Y.; Luo W.; Wang C.; Lacey S. D.; Dai J.; et al. Toward garnet electrolyte-based Li metal batteries: An ultrathin, highly effective, artificial solid-state electrolyte/metallic Li interface. Sci. Adv. 2017, 3 (4), e1601659 10.1126/sciadv.1601659. 28435874 PMC5384807

[ref33] Luo W.; Gong Y.; Zhu Y.; Fu K. K.; Dai J.; Lacey S. D.; Wang C.; Liu B.; Han X.; Mo Y.; et al. Transition from superlithiophobicity to superlithiophilicity of garnet solid-state electrolyte. J. Am. Chem. Soc. 2016, 138 (37), 12258–12262. 10.1021/jacs.6b06777. 27570205

[ref34] Luo W.; Gong Y.; Zhu Y.; Li Y.; Yao Y.; Zhang Y.; Fu K.; Pastel G.; Lin C. F.; Mo Y.; et al. Reducing interfacial resistance between garnet-structured solid-state electrolyte and Li-metal anode by a germanium layer. Adv. Mater. 2017, 29 (22), 1606042 10.1002/adma.201606042. 28417487

[ref35] Fu K.; Gong Y.; Fu Z.; Xie H.; Yao Y.; Liu B.; Carter M.; Wachsman E.; Hu L. Transient behavior of the metal interface in lithium metal–garnet batteries. Angew. Chem., Int. Ed. 2017, 56 (47), 14942–14947. 10.1002/anie.201708637. 28994191

[ref36] Han X.; Gong Y.; Fu K. K.; He X.; Hitz G. T.; Dai J.; Pearse A.; Liu B.; Wang H.; Rubloff G.; et al. Negating interfacial impedance in garnet-based solid-state Li metal batteries. Nat. Mater. 2017, 16 (5), 572–579. 10.1038/nmat4821. 27992420

[ref37] Wang C.; Gong Y.; Liu B.; Fu K.; Yao Y.; Hitz E.; Li Y.; Dai J.; Xu S.; Luo W.; et al. Conformal, nanoscale ZnO surface modification of garnet-based solid-state electrolyte for lithium metal anodes. Nano Lett. 2017, 17 (1), 565–571. 10.1021/acs.nanolett.6b04695. 27936780

[ref38] Shao Y.; Wang H.; Gong Z.; Wang D.; Zheng B.; Zhu J.; Lu Y.; Hu Y.-S.; Guo X.; Li H.; et al. Drawing a soft interface: An effective interfacial modification strategy for garnet-type solid-state Li batteries. ACS Energy Lett. 2018, 3 (6), 1212–1218. 10.1021/acsenergylett.8b00453.

[ref39] Zhu Y.; He X.; Mo Y. Strategies based on nitride materials chemistry to stabilize Li metal anode. Advanced Science 2017, 4 (8), 1600517 10.1002/advs.201600517. 28852614 PMC5566245

[ref40] Xue W.; Yang Y.; Yang Q.; Liu Y.; Wang L.; Chen C.; Cheng R. The effect of sintering process on lithium ionic conductivity of Li 6.4 Al 0.2 La 3 Zr 2 O 12 garnet produced by solid-state synthesis. RSC Adv. 2018, 8 (24), 13083–13088. 10.1039/C8RA01329B. PMC9079727 35542504

[ref41] Allen J. L.; Wolfenstine J.; Rangasamy E.; Sakamoto J. Effect of substitution (Ta, Al, Ga) on the conductivity of Li7La3Zr2O12. J. Power Sources 2012, 206, 315–319. 10.1016/j.jpowsour.2012.01.131.

[ref42] Rangasamy E.; Wolfenstine J.; Sakamoto J. The role of Al and Li concentration on the formation of cubic garnet solid electrolyte of nominal composition Li7La3Zr2O12. Solid State Ionics 2012, 206, 28–32. 10.1016/j.ssi.2011.10.022.

[ref43] Shimonishi Y.; Toda A.; Zhang T.; Hirano A.; Imanishi N.; Yamamoto O.; Takeda Y. Synthesis of garnet-type Li7– xLa3Zr2O12– 1/2x and its stability in aqueous solutions. Solid State Ionics 2011, 183 (1), 48–53. 10.1016/j.ssi.2010.12.010.

[ref44] Yang H.-C.; Hou J.; Chen V.; Xu Z.-K. Surface and interface engineering for organic–inorganic composite membranes. Journal of Materials Chemistry A 2016, 4 (25), 9716–9729. 10.1039/C6TA02844F.

[ref45] Cheng X. B.; Hou T. Z.; Zhang R.; Peng H. J.; Zhao C. Z.; Huang J. Q.; Zhang Q. Dendrite-free lithium deposition induced by uniformly distributed lithium ions for efficient lithium metal batteries. Advanced materials 2016, 28 (15), 2888–2895. 10.1002/adma.201506124. 26900679

[ref46] Xiang J.; Yuan L.; Shen Y.; Cheng Z.; Yuan K.; Guo Z.; Zhang Y.; Chen X.; Huang Y. Improved Rechargeability of Lithium Metal Anode via Controlling Lithium-Ion Flux. Adv. Energy Mater. 2018, 8 (36), 1802352 10.1002/aenm.201802352.

[ref47] Guo Y.; Ouyang Y.; Li D.; Wei Y.; Zhai T.; Li H. PMMA-assisted Li deposition towards 3D continuous dendrite-free lithium anode. Energy Storage Materials 2019, 16, 203–211. 10.1016/j.ensm.2018.05.012.

[ref48] Guan X.; Wang A.; Liu S.; Li G.; Liang F.; Yang Y. W.; Liu X.; Luo J. Controlling nucleation in lithium metal anodes. Small 2018, 14 (37), 1801423 10.1002/smll.201801423. 30047235

[ref49] Liu Y.; Lin D.; Yuen P. Y.; Liu K.; Xie J.; Dauskardt R. H.; Cui Y. An artificial solid electrolyte interphase with high Li-ion conductivity, mechanical strength, and flexibility for stable lithium metal anodes. Adv. Mater. 2017, 29 (10), 1605531 10.1002/adma.201605531. 28032934

[ref50] Li X.; Kersey-Bronec F. E.; Ke J.; Cloud J. E.; Wang Y.; Ngo C.; Pylypenko S.; Yang Y. Study of lithium silicide nanoparticles as anode materials for advanced lithium ion batteries. ACS Appl. Mater. Interfaces 2017, 9 (19), 16071–16080. 10.1021/acsami.6b16773. 28453258

[ref51] Lang J.; Charlot J. Li3N–Si3N4 system. Rev. Chim. Miner. 1970, 7 (1), 121–131.

[ref52] Yamane H.; Kikkawa S.; Koizumi M. Preparation of lithium silicon nitrides and their lithium ion conductivity. Solid State Ionics 1987, 25 (2–3), 183–191. 10.1016/0167-2738(87)90119-6.

[ref53] Ulvestad A.; Mæhlen J. P.; Kirkengen M. Silicon nitride as anode material for Li-ion batteries: Understanding the SiNx conversion reaction. J. Power Sources 2018, 399, 414–421. 10.1016/j.jpowsour.2018.07.109.

[ref54] Li Y.; Hirosaki N.; Xie R.; Takeka T.; Mitomo M. Crystal, electronic structures and photoluminescence properties of rare-earth doped LiSi2N3. J. Solid State Chem. 2009, 182 (2), 301–311. 10.1016/j.jssc.2008.10.031.

[ref55] Houmes J. D.; zur Loye H.-C. Microwave synthesis of ternary nitride materials. J. Solid State Chem. 1997, 130 (2), 266–271. 10.1006/jssc.1997.7303.

[ref56] Wen Z.; Wang K.; Chen L.; Xie J. A new ternary composite lithium silicon nitride as anode material for lithium ion batteries. Electrochemistry communications 2006, 8 (8), 1349–1352. 10.1016/j.elecom.2006.06.022.

[ref57] Hashim U.; Chong S.W.; Liu W.-W. Fabrication of silicon nitride ion sensitive field-effect transistor for pH measurement and DNA immobilization/hybridization. J. Nanomater. 2013, 2013, 542737 10.1155/2013/542737.

[ref58] Zeuner M.; Pagano S.; Schnick W. Nitridosilicates and oxonitridosilicates: from ceramic materials to structural and functional diversity. Angew. Chem., Int. Ed. 2011, 50 (34), 7754–7775. 10.1002/anie.201005755. 21774043

[ref59] Raghavan R. Synthesis and electrochemical characterization of Silicon clathrates as anode materials for Lithium ion batteries. M.S. Thesis, Arizona State University, 2013.

[ref60] Yamashita T.; Kuwano S.; Kuriyama K.; Kushida K. Optical band gap of Li8SiN4 with disordered structure as a cathode material of lithium secondary batteries. physica status solidi (c) 2015, 12 (6), 845–848. 10.1002/pssc.201400214.

[ref61] Xu H.; Li Y.; Zhou A.; Wu N.; Xin S.; Li Z.; Goodenough J. B. Li3N-Modified garnet electrolyte for all-solid-state lithium metal batteries operated at 40° C. Nano Lett. 2018, 18 (11), 7414–7418. 10.1021/acs.nanolett.8b03902. 30352159

[ref62] Chen K.; Pathak R.; Gurung A.; Adhamash E. A.; Bahrami B.; He Q.; Qiao H.; Smirnova A. L.; Wu J. J.; Qiao Q.; et al. Flower-shaped lithium nitride as a protective layer via facile plasma activation for stable lithium metal anodes. Energy Storage Mater. 2019, 18, 389–396. 10.1016/j.ensm.2019.02.006.

[ref63] Takeda Y.; Yamamoto O.; Imanishi N. Lithium dendrite formation on a lithium metal anode from liquid, polymer and solid electrolytes. Electrochemistry 2016, 84 (4), 210–218. 10.5796/electrochemistry.84.210.

