# Supplementary material for: Mitigating Interfacial Mismatch between Lithium Metal and Garnet-Type Solid Electrolyte by Depositing Metal Nitride Lithiophilic Interlayer
Source: ACS Appl Energy Mater. 2022 Jan 7;5(1):648–57. doi: 10.1021/acsaem.1c03157 (PMC8790721; doi:10.1021/acsaem.1c03157)
Supplement: Supplementary file 1 — ae1c03157_si_001.pdf [file ae1c03157_si_001.pdf]

Supporting Information

Mitigating interfacial mismatch between lithium metal and garnet-type solid electrolyte by depositing metal nitride lithiophilic interlayer

Abiral Baniya[1], *Ashim Gurung*[2], *Jyotshna Pokharel*[2], *Ke Chen*[2], *Rajesh Pathak*[3], *Buddhi Sagar Lamsal*[2], *Nabin Ghimire*[2], *Raja Sekhar Bobba*[1], *Sheikh Ifatur Rahman*[2], *Sally Mabrouk*[1], *Alevtina L. Smirnova*[4], *Kang Xu*[5], *Quinn Qiao*[1*]

[1]Mechanical and Aerospace Engineering, Syracuse University, Syracuse, NY 13244, USA

[2]Department of Electrical Engineering and Computer Science, South Dakota State University, Brookings, SD 57007, USA

[3]Applied Materials Division, Argonne National Laboratory, Lemont, IL, 60439, USA

[4]Department of Chemistry and Applied Biological Sciences, South Dakota School of Mines and Technology, Rapid City, SD 57701, USA

[5] Battery Science Branch, Sensor and Electron Devices Directorate, U.S. Army Research Laboratory, Adelphi, MD, 20783, USA

*Correspondence: quqiao@syr.edu

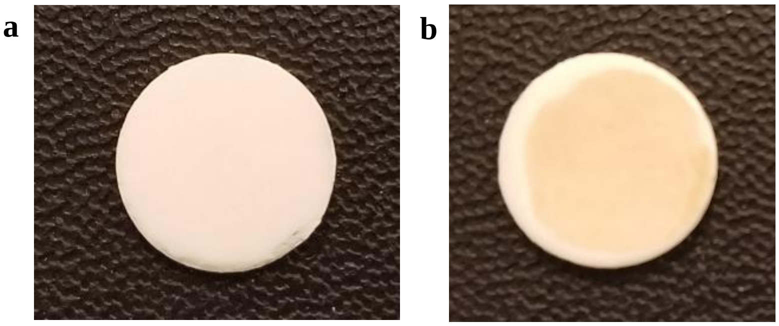

Figure S1. Digital image of as -prepared (a) bare Al-LLZO of around 1mm thickness (b) 30 nm Si_3N_4 coated Al-LLZO, solid state electrolyte ceramic pellets

a

Surface	Cross-section	Sintering Temperature	Relative Density
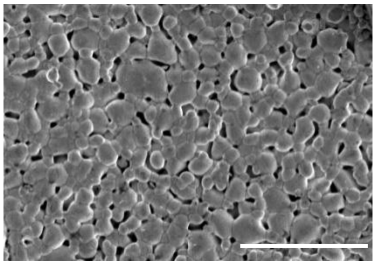	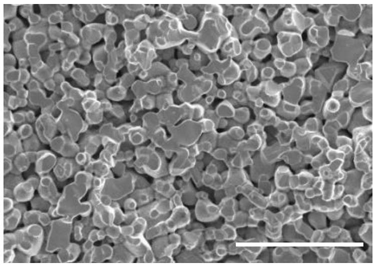	1100 ^{o}C	80%
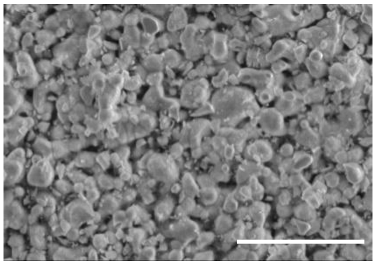	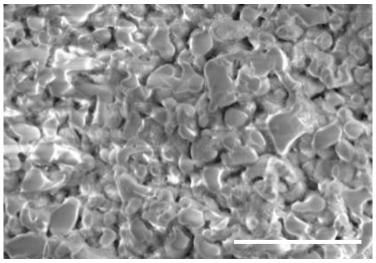	1230 ^{o}C	87.5%
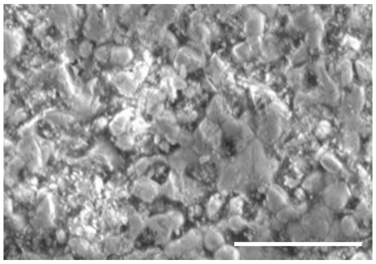	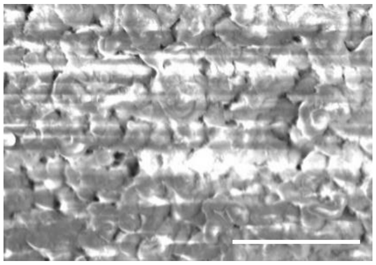	1250 ^{o}C	90.2%

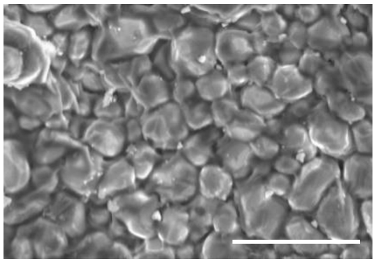	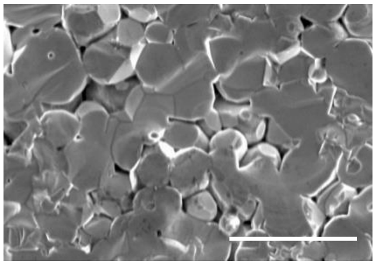	1280 °C	92%

b

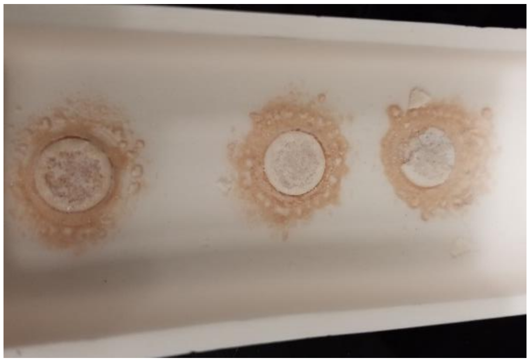

c

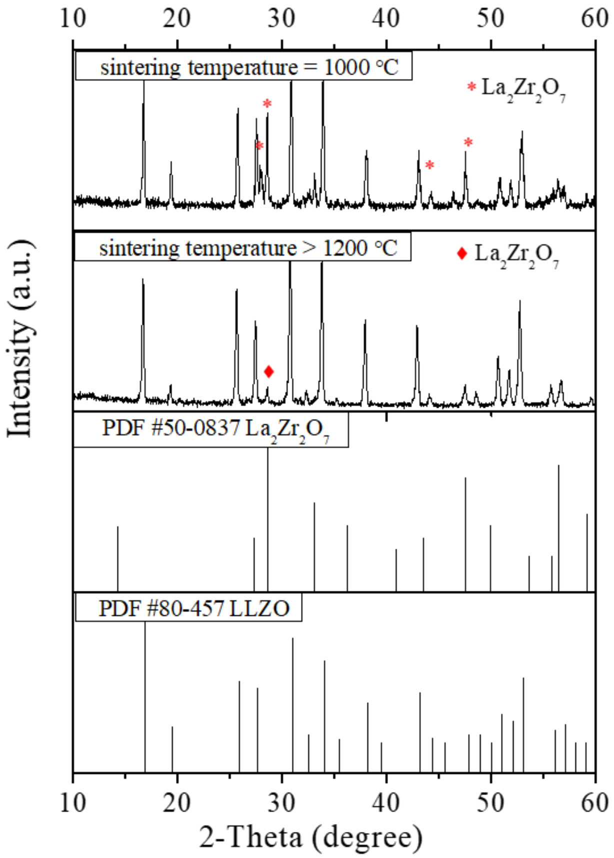

10 20 30 40 50 60
sintering temperature = 1000 °C
* $La_2Zr_2O_7$
sintering temperature > 1200 °C
♦ $La_2Zr_2O_7$
PDF #50-0837 $La_2Zr_2O_7$
PDF #80-457 LLZO
Intensity (a.u.)
2-Theta (degree)

d

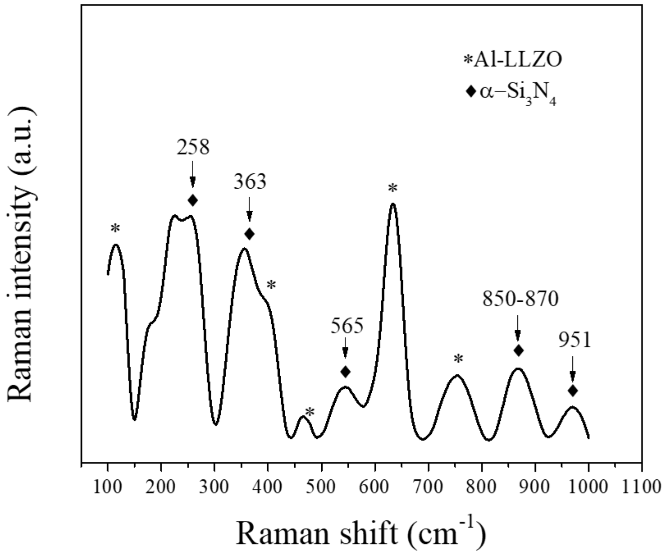

Figure S2. Different sintering temperatures were applied for the purpose of obtaining densified pellets. SEM analysis (a) Surface and cross-section SEM images of Al-LLZO ceramic disc at sintering temperature ranging from 1100- 1280 °C, sintered for an hour. The scale bars are 20 µm (b) Pellet melts and attaches to the crucible above 1280 °C. (c) XRD showing removal of impurity phase $La_2Zr_2O_7$ from solid electrolyte pellet after increasing the sintering temperature to 1200 ℃.[1] (d) Raman mapping of Al-LLZO and Si_3N_4 thin film deposited on Al-LLZO.

When sintering temperature is low around 1000 ℃, the XRD (Figure S2c) shows formation of impurity phase such as $La_2Zr_2O_7$ (equation(1)). However, when temperature is increased to 1200 ℃, as described in [1], Li_2O reacts with $La_2Zr_2O_7$ to form LLZO again as shown in equation (2). This is also justified by the disappearance of $La_2Zr_2O_7$ impurity phase XRD peaks after sintering above 1200 ℃ in Figure S2c.

$$Li_2CO_3+La_2O_3+2ZrO_2 \longrightarrow Li_2O+La_2Zr_2O_7+CO_2 \quad (1)$$

$$7Li_2CO_3+3La_2O_3+4ZrO_2 \longrightarrow 2Li_7La_3Zr_2O_{12}+7CO_2 \quad (2)$$

Similarly, figure S2d shows the Raman spectra of Si_3N_4 thin film deposited on top of Al-LLZO solid electrolyte. The peaks at 258, 363, 565, 850-870, and 951 cm^{-1} denoted by ♦ are characteristic of α- phase crystalline Si_3N_4 [2]. The remaining peaks denoted by * are characteristic of cubic phase Al-LLZO [3].

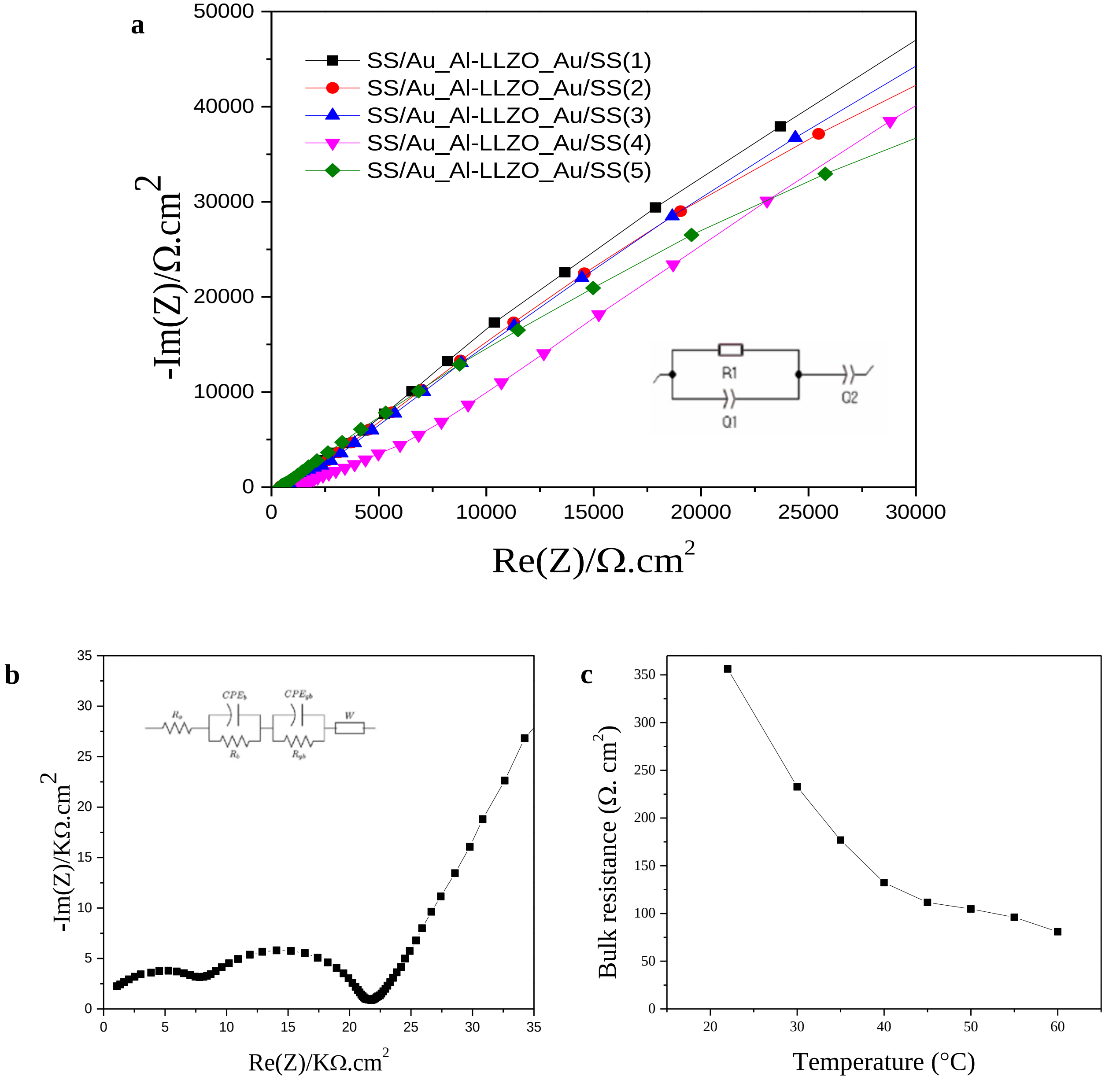

Figure S3. EIS spectra of (a) Al-LLZO pellets sintered at 1280°C with Au as blocking electrodes for 5 different coin cell samples, (b) Al-LLZO pellets sintered at lower temperature of 1100°C. (c) Bulk resistance response to change in temperature of Al-LLZO pellets sintered at 1280°C for an hour. The insets show the equivalent circuit for thus obtained EIS results.

As observed in Figure S3a when pellets were sintered at 1280 °C the Nyquist plot shows mainly the bulk response whereas the grain boundary contribution appears negligible compared to samples sintered at temperature < 1100 °C (Figure S3b) with two clearly visible semicircles indicating presence of significant grain boundary resistance.

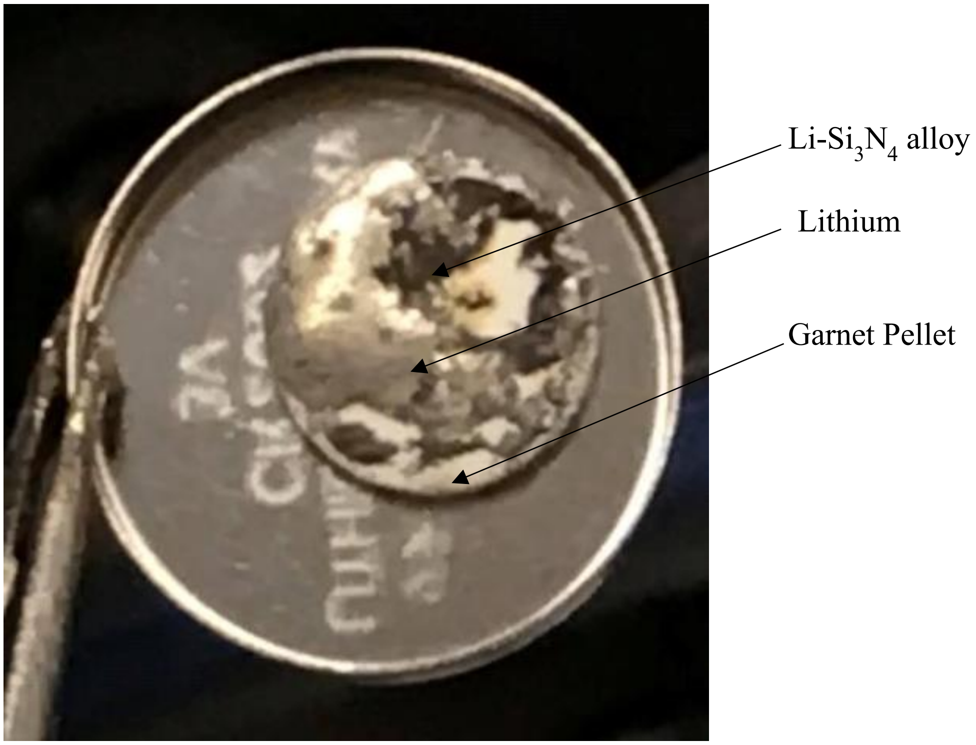

Figure S4. Lithiated Si_3N_4-coated Al-LLZO garnet SSE pellet. The Si_3N_4 coating becomes dark in color as shown above. These lithiated pellets were used to perform XRD to observe if new peaks arise due to formation of Li-Si_3N_4 alloy. New peaks that were assigned to lithium silicon (JCPDS #40-1449) were observed when compared with bare garnet solid electrolytes.

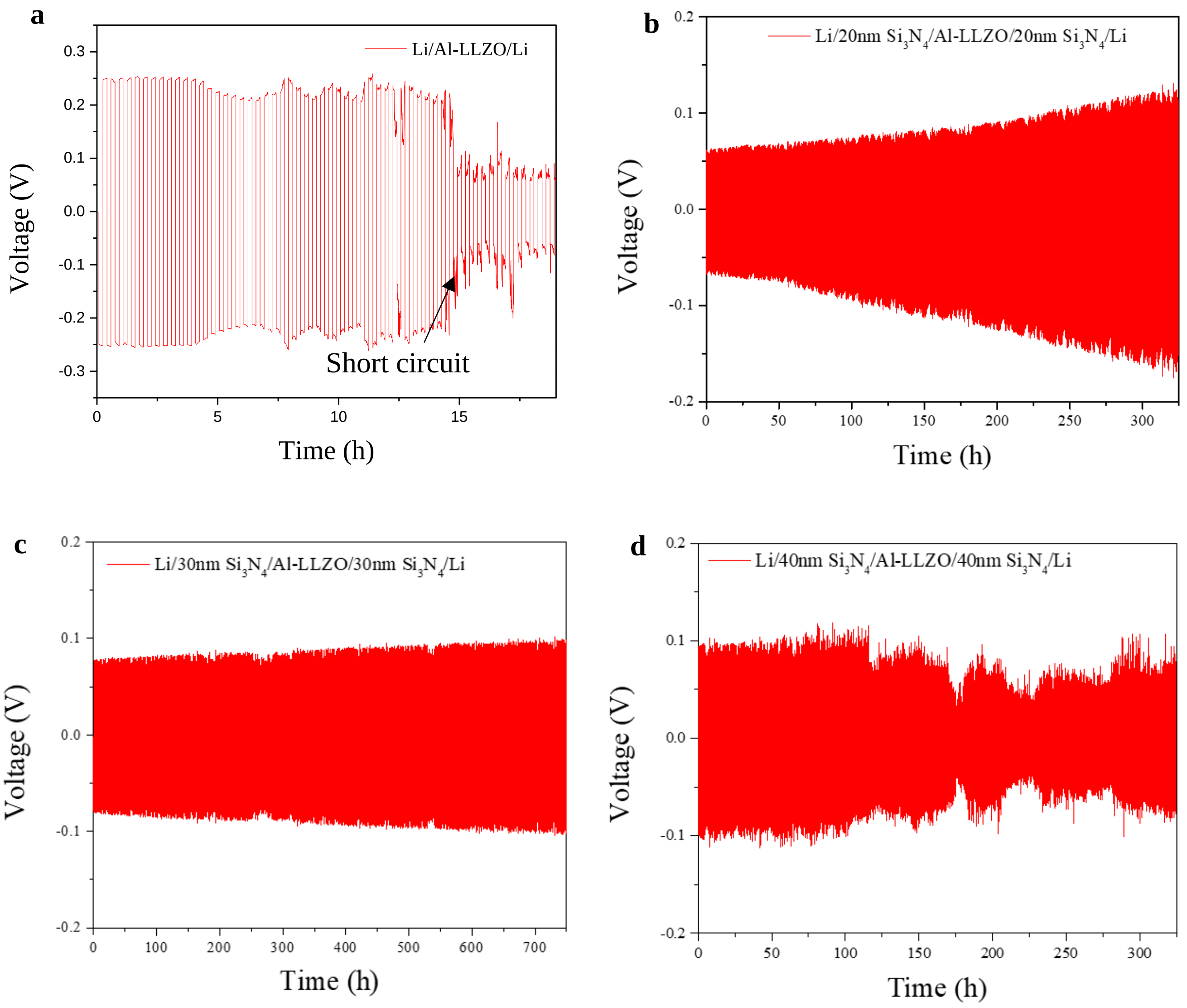

Figure S5. Optimization of interlayer thickness by evaluating the cycling stability of Li/garnet/Li symmetrical cells at 0.1 mA cm^{-2}, Si_3N_4 thickness from top-left to right are: a) 0 nm (bare garnet), the cell short circuits after 15 hours of cycling. b) 20 nm, plating/stripping cycles becomes unstable with rapid increase in overpotential voltage after 150 hours of cycling c) 30nm, cycling is stable for more than 700 hours and d) 40 nm, the cell shows increased voltage polarization and fluctuations after 110 hours .

The Li-symmetrical cells for optimizing Si_3N_4 thickness were cycled under constant current density of 0.1 $mAcm^{-2}$. As shown in Figure S5a, the symmetrical cell with bare garnet short circuited after only 15 hours of cycling. In comparison, when 20 nm of Si_3N_4 interlayer was introduced (Figure S5b), the plating/stripping cycles become very stable until 150 hours. While

30nm deposited Si_3N_4 interlayer (Figure S5c) shows the optimum stable plating/stripping cycles for more than 700 hours, where further increase in thickness of Si_3N_4 interlayer to 40 nm limited the cycling to 110 hours (Figure S5d). Further analysis of data on cycling of the symmetrical cell assembled with 40 nm Si_3N_4 modified LLZO shows that this is not a self-healing phenomenon of the LLZO electrolyte as reported in some literature. Instead, it shows increased voltage polarization and voltage fluctuations during lithium deposition/ dissolution which indicates cell failure as explained in literature [4-7].

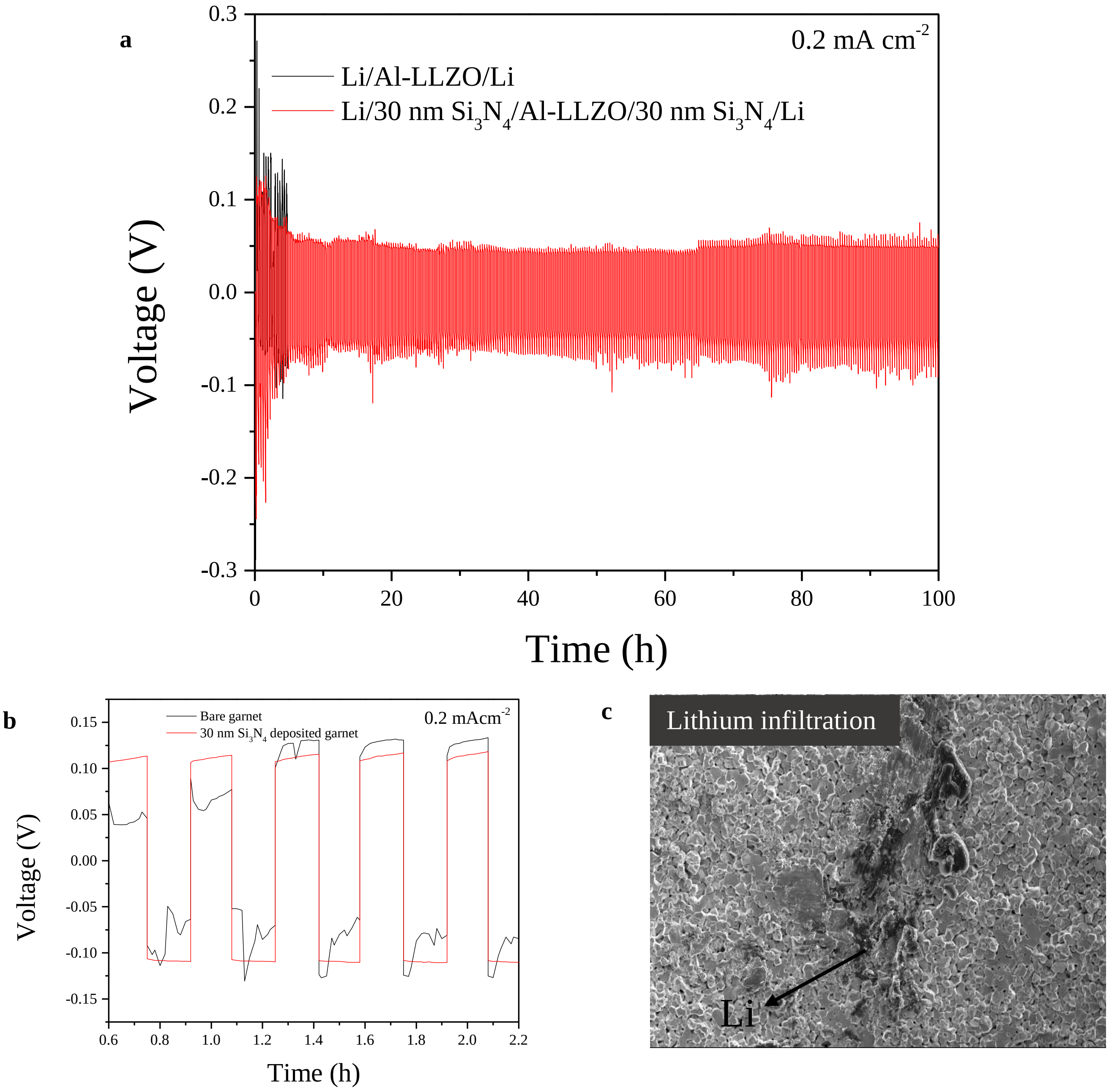

Figure S6. Galvanostatic plating/ stripping cycles of (a) 30 nm Si_3N_4 deposited garnet SSE at 0.2 mA cm^{-2}, 22°C compared with that for bare garnet, (b) First few cycles showing stable plating at 0.2 mA cm^{-2} for 30nm Si_3N_4 deposited garnet whereas unstable plating for bare garnet (c) Cross-section SEM of short-circuited symmetrical cells after galvanostatic cycling showing Li infiltration in SSE garnet pellet.

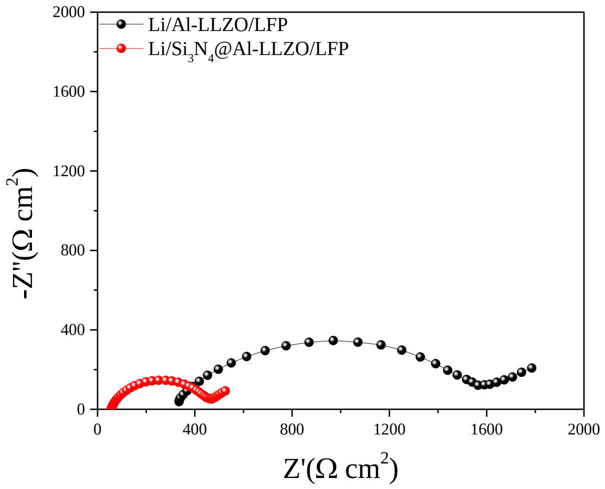

Figure S7. Nyquist plot of full cells with bare garnet and Si_3N_4 modified garnet.

As shown in the Nyquist plots (Figure S7), the total area specific resistance of full cell with Si_3N_4 modified Al-LLZO garnet electrolyte is < 500 Ωcm^2 versus ~1600 Ωcm^2 with bare garnet at room temperature.

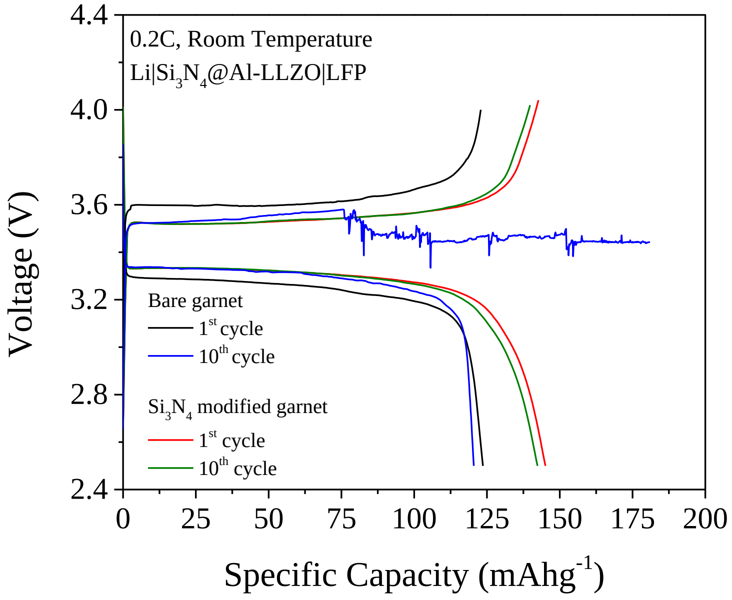

Figure S8. Comparison of full cell performance between bare garnet and Si_3N_4 modified garnet.

As depicted in Figure S8, the full cell with bare Al-LLZO garnet electrolyte displayed larger overpotential compared to Si_3N_4 modified Al-LLZO garnet and unstable charge voltage curves at 10th cycle, suggesting short-lived cycling.

Table S1: Performance comparison of different nitride and other interlayers for reducing interfacial resistance at Li/solid electrolyte interface. (SE = Solid Electrolyte, CCD = Critical Current Density, LATP = $Li_{1.3}Al_{0.3}Ti_{1.7}(PO_4)_3$, LPCVD = low-pressure chemical vapor deposition, LLZTO = $Li_{6.5}La_3Zr_{1.5}Ta_{0.5}O_{12}$, Ebeam = electron beam thermal deposition, PEO = poly (ethylene oxide), PVC-TPU = poly (vinylene carbonate)- thermoplastic polyurethanes, LAGP = $Li_{1.5}Al_{0.5}Ge_{1.5}(PO_4)_3$, ITO = indium tin oxide, Al-LLZO = $Li_{6.25}Al_{0.25}La_3Zr_2O_{12}$.)

Coating material	Solid electrolyte	Interlayer thickness	Interface ASR (Ω cm^2)	Operating temperature (℃)	SE thickness	Voltage Overpotential/ Current density (mV/ mAcm^{-2})	CCD (mA cm^{-2})	Method	Reference
Boron nitride	LATP with liquid electrolyte wetting	1-2 µm	3×10^4	60	0.5-1 mm	156 / 0.3	0.5	LPCVD	[8]
Lithium nitride	LLZTO	N/A	~120	40	N/A	60 / 0.1	0.2	Ebeam	[9]
Boron nitride nanosheet	PEO-based	150 nm	~100	60	~ 40 µm	100 / 0.3	0.3	Chemical exfoliation	[10]
ITO	LLZTO	40 nm	32	30	N/A	20 / 0.1	1.05	Magnetron Sputtering	[11]
Candle soot	LLZTO	1.5 µm	50	60	~ 1mm	28 / 0.1	0.6	Flame vapor deposition	[12]
PVC-TPU	LAGP	50 µm	~ 890	25	N/A	100 / 0.1	0.5	Drop casting	[13]
**Silicon nitride**	**Al-LLZO**	**30 nm**	**84.5**	**22**	**1 mm**	**80 / 0.1**	**1**	**RF sputtering**	**This work**

References

1. Liu, G., T. Li, Y. Xing, and W. Pan. *Synthesis of Li7La3Zr2O12 Solid Electrolyte by Solid Phase Sintering Method*. in *IOP Conference Series: Materials Science and Engineering*. 2019. IOP Publishing.
2. ACOSTA-ENRIQUEZ, E., M. ACOSTA-ENRIQUEZ, R. CASTILLO-ORTEGA, M. ZAYAS, and M. PECH-CANUL, *NANOSTRUCTURED FIBERS OF A-SI3N4 DEPOSITED BY HYSY-CVD.*
3. Dhivya, L., K. Karthik, S. Ramakumar, and R. Murugan, *Facile synthesis of high lithium ion conductive cubic phase lithium garnets for electrochemical energy storage devices.* RSC advances, 2015. **5**(116): p. 96042-96051.
4. Li, H., W. Liu, X. Yang, J. Xiao, Y. Li, L. Sun, X. Ren, P. Zhang, and H. Mi, *Fluoroethylene carbonate-Li-ion enabling composite solid-state electrolyte and lithium metal interface self-healing for dendrite-free lithium deposition.* Chemical Engineering Journal, 2021. **408**: p. 127254.
5. Xiong, S., Y. Liu, P. Jankowski, Q. Liu, F. Nitze, K. Xie, J. Song, and A. Matic, *Design of a Multifunctional Interlayer for NASCION-Based Solid-State Li Metal Batteries.* Advanced Functional Materials, 2020. **30**(22): p. 2001444.
6. Xia, S., J. Lopez, C. Liang, Z. Zhang, Z. Bao, Y. Cui, and W. Liu, *High-Rate and Large-Capacity Lithium Metal Anode Enabled by Volume Conformal and Self-Healable Composite Polymer Electrolyte.* Advanced Science, 2019. **6**(9): p. 1802353.
7. Feng, Y., C. Zhang, B. Li, S. Xiong, and J. Song, *Low-volume-change, dendrite-free lithium metal anodes enabled by lithophilic 3D matrix with LiF-enriched surface.* Journal of Materials Chemistry A, 2019. **7**(11): p. 6090-6098.
8. Cheng, Q., A. Li, N. Li, S. Li, A. Zangiabadi, W. Huang, A.C. Li, T. Jin, Q. Song, and W. Xu, *Stabilizing solid electrolyte-anode interface in Li-metal batteries by boron nitride-based nanocomposite coating.* Joule, 2019. **3**(6): p. 1510-1522.
9. Xu, H., Y. Li, A. Zhou, N. Wu, S. Xin, Z. Li, and J.B. Goodenough, *Li3N-modified garnet electrolyte for all-solid-state lithium metal batteries operated at 40 C.* Nano letters, 2018. **18**(11): p. 7414-7418.
10. Shen, B., T.-W. Zhang, Y.-C. Yin, Z.-X. Zhu, L.-L. Lu, C. Ma, F. Zhou, and H.-B. Yao, *Chemically exfoliated boron nitride nanosheets form robust interfacial layers for stable solid-state Li metal batteries.* Chemical Communications, 2019. **55**(53): p. 7703-7706.
11. Lou, J., G. Wang, Y. Xia, C. Liang, H. Huang, Y. Gan, X. Tao, J. Zhang, and W. Zhang, *Achieving efficient and stable interface between metallic lithium and garnet-type solid electrolyte through a thin indium tin oxide interlayer.* Journal of Power Sources, 2020. **448**: p. 227440.
12. Zhang, Y., J. Meng, K. Chen, Q. Wu, X. Wu, and C. Li, *Behind the Candelabra: A Facile Flame Vapor Deposition Method for Interfacial Engineering of Garnet Electrolyte To Enable Ultralong Cycling Solid-State Li–FeF3 Conversion Batteries.* ACS Applied Materials & Interfaces, 2020. **12**(30): p. 33729-33739.
13. Zhai, P., L. Fu, S. Yuan, L. Shi, J. Zhu, Y. Zhao, and Z. Wang, *Ionic Conductive Thermoplastic Polymer Welding Layer for Low Electrode/Solid Electrolyte Interface Resistance.* ACS Applied Energy Materials, 2020. **3**(7): p. 7011-7019.
